# DNA repair and aging: the impact of the p53 family

**DOI:** 10.18632/aging.100858

**Published:** 2015-12-11

**Authors:** Sara Nicolai, Antonello Rossi, Nicola Di Daniele, Gerry Melino, Margherita Annicchiarico-Petruzzelli, Giuseppe Raschellà

**Affiliations:** ^1^ Department of Experimental Medicine and Surgery, University of Rome “Tor Vergata”, 00133 Rome, Italy; ^2^ Medical Research Council, Toxicology Unit, Hodgkin Building, Leicester University, Leicester LE1 9HN, UK; ^3^ Biochemistry Laboratory, IDI-IRCCS, Rome 00100, Italy; ^4^ ENEA Research Center Casaccia, Laboratory of Biosafety and Risk Assessment, 00123 Rome, Italy

**Keywords:** aging, DNA repair, p53, p63, p73, homologous recombination, Non homologous end joining

## Abstract

Cells are constantly exposed to endogenous and exogenous factors that threaten the integrity of their DNA. The maintenance of genome stability is of paramount importance in the prevention of both cancer and aging processes. To deal with DNA damage, cells put into operation a sophisticated and coordinated mechanism, collectively known as DNA damage response (DDR). The DDR orchestrates different cellular processes, such as DNA repair, senescence and apoptosis. Among the key factors of the DDR, the related proteins p53, p63 and p73, all belonging to the same family of transcription factors, play multiple relevant roles. Indeed, the members of this family are directly involved in the induction of cell cycle arrest that is necessary to allow the cells to repair. Alternatively, they can promote cell death in case of prolonged or irreparable DNA damage. They also take part in a more direct task by modulating the expression of core factors involved in the process of DNA repair or by directly interacting with them. In this review we will analyze the fundamental roles of the p53 family in the aging process through their multifaceted function in DDR.

## The aging process

Aging can be defined as the gradual biological impairment of normal body functions accompanied by a decreased ability to respond to stress and by a greatly increased risk of morbidity and mortality [1]. This complex and multi-factorial process is characterized by a progressive failure in maintaining tissue homeostasis with a consequent direct impact on the functional ability of organs and eventually of the entire organism that causes a significant loss of fitness. The resulting deterioration represents the main risk factor for the age-related pathologies, including cancer, cardiovascular diseases and neurodegenerative disorders that ultimately lead aged organisms to death.

The full understanding of aging is far from being achieved, due to the multiplicity of mechanisms involved in this process. However, in the last decades, the ever-growing number of analytical techniques has allowed a better knowledge of the pathways underlying this process and a deeper understanding of the molecular basis of aging with the far-reaching goal of extending the human life span. Despite the existence of different but not mutually exclusive theories on aging (see [2] for an overview), it is now widely accepted that the main cause of this process is the gradual, life-long accumulation of molecular and cellular damage [1,3,4] .

Recently, Lopez-Otìn and co-workers have exhaustively reviewed the cellular and molecular hallmarks of aging [5], precisely defining the so-called “aging phenotype”. In particular, they classified these hallmarks into three different categories: 1- primary hallmarks, such as DNA damage, telomere loss and epigenetic alterations that initially trigger the aging process; 2- antagonistic hallmarks, as senescence or Reactive Oxygen Species (ROS), whose effects initially protect the organism from damage, but become progressively negative throughout the process promoted by the primary hallmarks; 3- integrative hallmarks, like inflammation or stem cell exhaustion, that directly impair homeostasis when the process that leads to accumulation of damage becomes irreversible.

Genomic instability is considered one of the main drivers of the aging process [6]. Indeed, during the cell lifetime, the genomic DNA is continuously exposed to different hazards that undermine its integrity and functionality. In fact, DNA can be either challenged by exogenous environmental factors, such as oxidative stress, genotoxic drugs and ionizing radiation [7–11] or by endogenous chemicals, the most relevant of which are ROS, by-products of the normal mitochondrial metabolism [12–14] that can also cause cellular apoptosis by p53 activation [15]. Furthermore, replication errors and spontaneous hydrolysis reactions represent additional endogenous sources of DNA damage [16].

**Figure 1:**
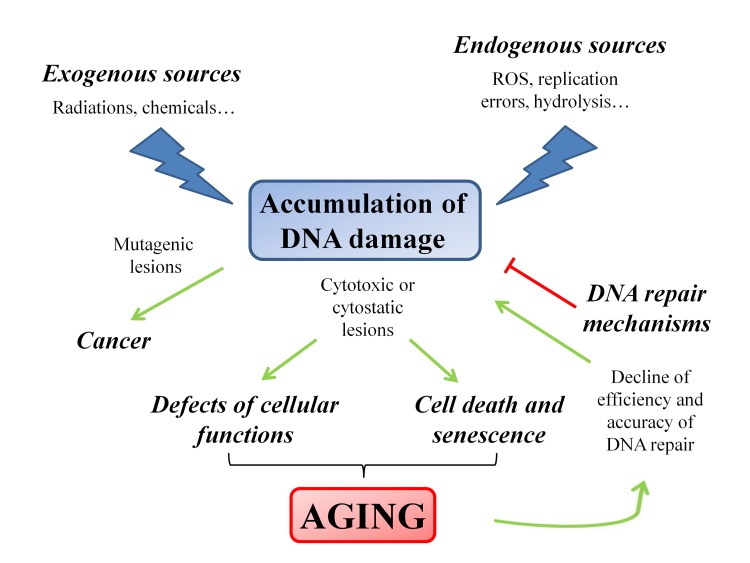
Schematic representation of the factors and the pathways that modulate the aging process The accumulation of DNA damage plays a pivotal role in triggering the aging process. The progressive failure of the efficiency of the DNA repair mechanisms induces a feedback loop that enforces the aging process.

Different cellular outcomes can follow DNA injury: if DNA lesions are not properly repaired or are fixed by replication, they are converted into permanent mutations that significantly increase the risk of cancer; alternatively, DNA damage could also cause replication arrest leading to cellular senescence or cell death, thus contributing to the onset of the aging process. Therefore, cancer and aging, both arising as the consequence of irreparable DNA damage, can be considered as the two sides of the same coin [13]. Taking into account these considerations, little doubt remains about the causative role of DNA damage in tumorigenesis as well as in aging [17]. In particular, in aging the increase of DNA damage rate is the overall result of the imbalance between generation and disposal of by-products deriving from cellular metabolism [18], and the functional decline of DNA repair efficiency [19–21] (Figure 1).

The multi-factorial nature of aging has somehow hampered the study of this process in complex organisms in part because of the lack of genetically-modified animal models carrying multiple mutations that mimic the progressive functional deterioration typical of the aged organisms. Some age-related pathologies such as Alzheimer's disease give some hints to understand the multifactorial nature of the aging process [22]. Furthermore, the pivotal role of the maintenance of genome stability in preventing the aging process through the activation of different DNA repair mechanisms is suggested by progeroid syndromes such as Werner syndrome, Hutchinson–Gilford progeria syndrome and Cockayne syndrome [23,24]. These well-defined diseases share, in fact, defects in some of the key components of the cellular response to DNA damage and are characterized by an early onset of aging-related features in affected individuals. Thus, progeroid syndromes provide important clues to understand the molecular mechanisms underlying human aging and, despite their “segmental” nature, represent a useful tool to improve our knowledge on still unknown aspects of the aging process. In addition, although unsuitable for representing complete *in vivo* models, single gene knock-outs have helped in clarifying the aging-related role of individual genes, for instance of those belonging to the p53 family [25–32]. In the future, recently acquired techniques such as CRISPR/Cas9 that allows the ablation of multiple genes in a single step [33,34], will likely have an impact in this field by providing more faithful experimental replica of aging in which the role of individual players of the process can be identified.

## The DNA repair mechanisms in mammals

**Figure 2:**
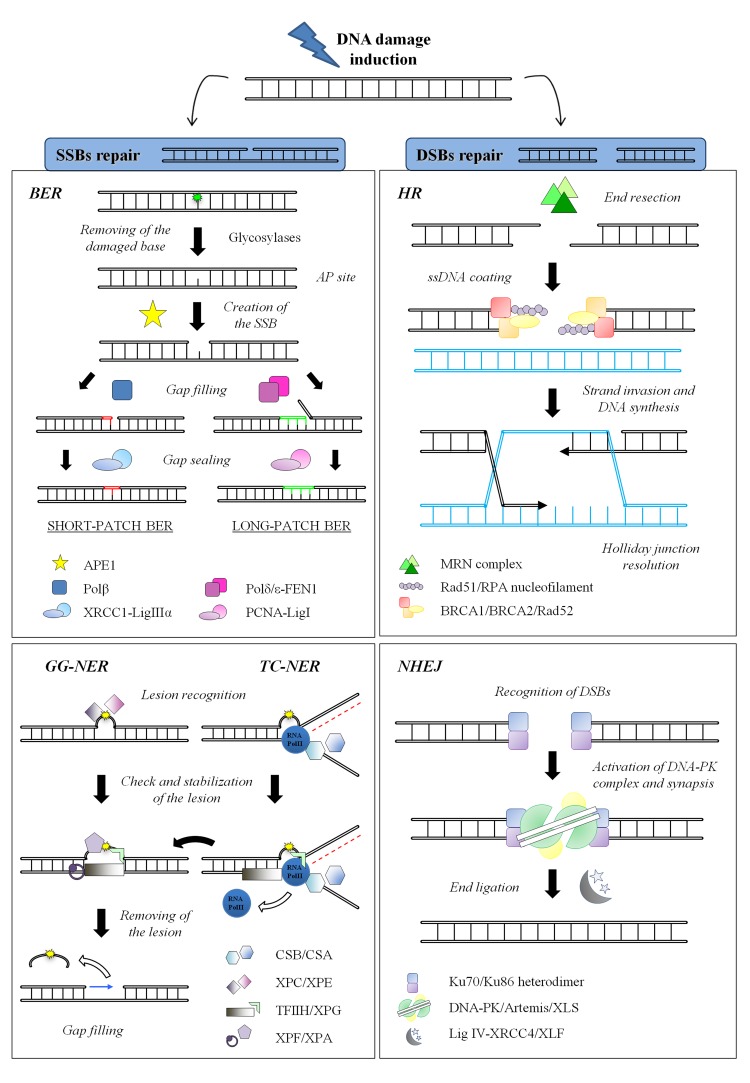
DNA repair systems in mammals. *(Left)* SSBs repair mechanisms: BER Different damage-specific glycosylases (such as OGG1 or UNG) recognize and excise the damaged base (here shown in *green*). The resulting AP site is target of the endonuclease APE1 that physically creates the SSB. In the short-patch sub-pathway, only one nucleotide is replaced by Polβ while the gap is sealed by XRCC1-ligase IIIα. On the other hand, during the long-patch sub-pathway, Polδ/ε synthesize 2-8 nucleotides and FEN1 removes the 5′ flap DNA, whereas the ligation step is carried out by the complex PCNA-ligase I; **NER**: The initial step of lesion detection (here shown in *yellow*) is the only difference between GG-NER and TC-NER and it is executed by XPC/XPE or by CSB/CSA, respectively. After this step, the DNA helix is locally opened by the helicases subunits of TFIIH that allow the damage verification by XPA. The endonucleases XPF and XPG finally perform a dual incision flanking the lesion, thus releasing a 25-30 nucleotides oligomer. The single-strand gap is filled by the polymerases δ and ε, while the final nick is sealed by DNA ligase I and the complex XRCC1-ligase IIIα. ***(Right) DSBs repair mechanisms*: HR**: This process is initiated by the resection of the DSB by the nuclease activity of the MRN complex, in order to generate 3′ ssDNA tails. The protruding DNA is rapidly coated by RPA protein to keep it unwound; then Rad51, supported by several other factors such as BRCA1, BRCA2 and Rad52, drives the formation of the nucleoprotein filament on the ssDNA coated with RPA. RAD51 also mediates the strand invasion and the search for homologous sequences of the nucleoprotein complex. The process ends with the resolution of Holliday junction; **NHEJ**: The heterodimer Ku70/Ku86 rapidly senses the presence and binds to free dsDNA ends, thus recruiting and activating the DNA-PK catalytic subunit. The kinase activity of DNA-PK is required to activate the nuclease activity of Artemis, necessary to process ends before joining. The Ligase IV and its cofactor XRCC4 finally perform the ligation step. XLF and XLS are recently established members of this pathway whose functions are still under debate.

To cope with DNA damage, cells have evolved the DNA Damage Response (DDR), an intricate and finely regulated genomic maintenance apparatus that senses and signals the presence of lesions to finally promote their repair. The DDR includes various checkpoints, signal-transduction cascades and effectors systems that affect important cellular processes, such as replication, transcription, cell cycle progression, chromatin remodeling, differentiation or apoptosis [35–38]. Furthermore, the DDR triggers distinct repair pathways, each specific for a particular class of DNA lesion aimed at restoring the integrity of the chemical structure of DNA molecule [39]. In Figure 2 the main repair mechanisms for Single Strand Breaks (SSBs) and Double Strand Breaks (DSBs) are schematized, including their key components. Taking into account all its properties, this complex network of DNA repair systems each one dedicated to a specific type of damage, is one of the most powerful determinant of the cell's choice among survival, replicative senescence or death [40,41].

## SSBs repair

Cells repair DNA lesions affecting only one strand of the double helix through excision mechanisms, divided into Base Excision Repair (BER) and Nucleotide Excision Repair (NER) (Figure 2 *left*). The mechanism of action shared by these two repair systems is based on the informational redundancy of two complementary strands in the genomic DNA. Hence, these “cut and patch” repair mechanisms recognize the damaged nucleotide, remove the damaged base and, by using the opposite strand as template, fill the gap and finally ligate the pieces. Either BER or NER are complex multi-step processes involving different sets of repair enzymes. In particular, the core complex of BER, mainly involved in repairing small DNA alterations such as the oxidative damage induced by ROS, is composed of some glycosylases that leave apurinic/apyrimidinic site (AP site), the endonuclease APE1, the polymerases β, δ and ε, the DNA ligase I and the complex XRCC1-ligase IIIα [42–44]. This mechanism is not directly linked to the aging process, probably because hereditary defects in the essential BER components are embryonically lethal [45,46]; nevertheless, multiple evidences demonstrate an age-related decline in BER efficiency that is most likely associated with the accumulation of oxidative lesions with aging [47–50].

On the contrary, NER, implicated in resolving most of the helix-distorting lesions such as the bulky-chemical adducts or the UV-induced photoproducts and the ionizing radiation-induced SSB [51], is divided into two sub-pathways: the global genome NER (GG-NER) and the transcription-coupled NER (TC-NER) that differ for the set of recognition proteins utilized (i.e. XPC and XPE for GG-NER; CSB and CSA for TC-NER) [52–55]. Following the initial recognition step, the two sub-pathways share the same core complex that is basically composed by the helicases subunits of TFIIH (XPB and XPD), the endonucleases ERCC1-XPF and XPG, the polymerases δ and ε, the DNA ligase I and the complex XRCC1-ligase IIIα [54,56]. NER is strictly connected with premature aging, as demonstrated by the association of deficiencies in this pathway with the human heritable disorders xeroderma pigmentosum (XP), trichothiodystrophy (TTD) and Cockayne syndrome (CS) [57]; moreover, several studies showed that NER efficiency and accuracy dramatically decline with age [58–60].

## DSBs repair

Breaks in both strands of DNA, as those caused by ionizing radiations, are the most lethal lesions that cells have to cope with: in fact, if left unrepaired, DSBs can lead to genomic rearrangements and eventually to cell transformation or to cell death. The two main cellular mechanisms to repair DSBs are the Homologous Recombination (HR) and the Non-Homologous End Joining (NHEJ) [61–63] (Figure 2 *right*). The HR pathway [64] provides an accurate repair of DSBs by using the sister chromatid as template. However, this mechanism is restricted to late S and G2 phases of the cell cycle, when an extra-copy of each chromosome is available, while its use seems to be inhibited in G1 phase, likely to prevent loss of heterozygosity [65–67]. The key factor of HR is the recombinase Rad51 that form the nucleoprotein filament implied in strand invasion and branch migration. Additional proteins involved in this process comprise the MRN complex (composed of its subunits Mre11, Rad50 and Nbs1), RPA, Rad52, BRCA1 and BRCA2 and other components whose role is still under scrutiny. Recently it has been demonstrated a decline in the expression levels of some HR proteins (such as Mre11, Rad51 and BRCA1 but not BRCA2) in aged murine and human oocytes that underlines the importance of active DNA repair in maintaining ovarian reserve [68,69].

The other main mechanism of DSBs repair, NHEJ, predominates in G0/G1 cells even if it works in all phases of cell cycle, thus representing the most utilized repair mechanism in eukaryotic cells [70]. This mechanism simply joins two broken ends by using extremely limited or none sequence homology [71,72]: This often results in little deletions or insertions that render NHEJ an error-prone process contributing to accumulation of mutations and aging [73]. The core protein components of NHEJ include the kinase DNA-PKcs, the Ku70-Ku86 heterodimer [74], the nuclease Artemis, the polymerases μ and λ, the DNA ligase IV and the scaffolding proteins XRCC4, XLF [75–77] and the newly characterized PAXX4/XLS/c9orf142 [78–80]. Depletion of PAXX4/XLS/c9orf142 in cells impaired DSB repair consistent with a defect in NHEJ [78]. During aging NHEJ becomes more error-prone and less efficient, as demonstrated in human senescent fibroblasts [81]; this decline is likely correlated with the altered regulation and the reduced availability of Ku proteins [82]. Furthermore, deficiency for other components of NHEJ pathway (such as XRCC4 and Ligase IV) leads to premature senescence and to a high degree of apoptosis; of note, these phenotypes are relieved in a p53^−/−^ background [83–86].

Finally, recent evidences revealed the existence of a third, less well-characterized DSBs repair pathway: the so-called microhomology-mediated end-joining (MMEJ) [87,88]. This mechanism can be considered as a sort of a hybrid between HR and NHEJ, as it joins broken ends with microhomology sequences of 5-25 base pairs in a Ku-independent manner. Even if it is an error-prone process, it could represent a backup mechanism that acts when other repair pathways fail.

The exact number of the components of each repair pathway is not known yet. Broadly, proteins that participate in the repair mechanisms can be subdivided in core proteins (i.e. those that are necessary to the execution of the process such as glycosylases, polymerases, ligases, etc.) and accessory proteins (i.e. those that facilitate and optimize the process but, whose presence is not indispensable [78]). Examples of accessory proteins are NR4A nuclear orphan receptors that interact with the DNA-PK catalytic subunit and, upon exposure to DNA damage, translocate to DSB foci by a mechanism dependent on the activity of poly(ADP-ribose) polymerase-1 (PARP-1) [89,90], APLF that functions together with PARP-3 to accelerate NHEJ [91] and Nucleolin which mediates nucleosome disruption critical for DNA double strand breaks repair [92].

A role apart among the accessory proteins is played by transcription factors that control the expression of essential components of the repair pathways. In their quality of transcriptional regulators, these proteins are not directly involved in the repair mechanisms but, they can greatly affect their execution by determining the availability of core components of the repair machinery [93]. In this regard, our laboratory has recently demonstrated the ability of the zinc finger factor ZNF281 in promoting the expression of XRCC2 and XRCC4 two proteins involved in HR and NHEJ, respectively [94].

Of note, recent work highlighted the presence of many transcription factors at the site of damaged DNA [95]. Interestingly, most of them were PARP-dependent for localization to sites of DNA damage. These latter findings expand the role of transcription factors in DNA repair since one of the most likely function of transcription factors in the proximity of damaged DNA is the recruitment of chromatin remodeling complexes, which can modulate the accessibility of core components of the DNA repair machinery.

In the third part of this review we will discuss the role that the transcription factors of the p53 family have in DNA repair and how this could be linked to the process of aging.

### The p53 family

**Figure 3:**
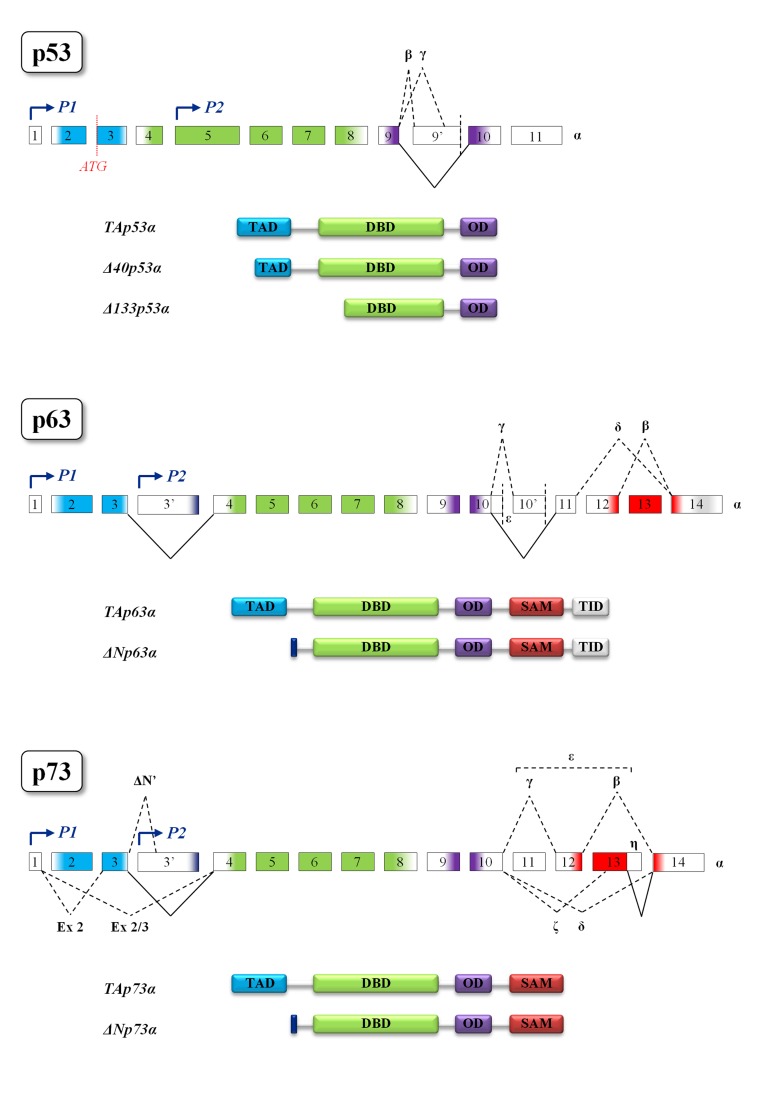
Structural motifs of p53 family members Gene and protein structures of p53 (top), p63 (middle) and p73 (bottom). Colors indicate protein domains encoded by the exons. The different transcription start sites P1 and P2 (indicated by blue arrows) give rise, respectively, to the TA and ∆N isoforms in both p63 and p73. In p53 gene the existence of an alternative translation start site (shown in red) generates the ∆40 isoform, while the ∆133 is transcribed by the P2 promoter. The multiple alternative splicing events leading to the different protein isoforms are represented by black dotted lines. Black solid lines represent splicing events leading to the formation of α proteins, the longest isoforms of each family member. Splicing of consecutive exons is omitted for simplicity. TAD, transactivation domain; DBD, DNA-binding domain; OD, oligomerization domain; SAM, sterile α-motif domain; TID, transcription inhibitory domain.

The p53 family is composed of a group of transcription factors, the well-characterized tumor suppressor p53, and its homologs p63 and p73 whose overall protein architecture is highly conserved from *Drosophila melanogaster* to humans [96]. The common gene framework of the p53 family is constituted by three main domains: an N-terminal transactivation domain (TAD), a central DNA-binding domain (DBD) and an oligomerization domain (OD). The sterile α-motif domain (SAM) involved in protein-protein interactions is distinctive of p63 and p73 and the C-terminal transcription inhibitory domain (TID) is only present in p63 [96]. Moreover, in p63 and p73 the usage of an internal promoter leads to the expression of two different types of proteins: the TA-isoforms, containing the whole N-terminal transactivation domain, and the truncated ∆N-isoforms, lacking almost all this domain. The ∆N-isoforms bound to their target promoters can behave as dominant-negative varieties of the TA-isoforms but, ΔNp63 can also affect the transcription of its target genes through the usage of alternative transcription activation domains [97]. It should also be mentioned that p53 is transcribed by an alternative p53 promoter in intron 4 and encodes N-terminal truncated forms whose function is to antagonize p53-dependent apoptosis or to inhibit cell replicative senescence [98,99]. In addition, alternative splicing mechanisms generate a great number of protein variants containing different C- and N-termini [96, 97, 100, 101] (Figure 3). The high sequence homology among p53 family members allows binding and transactivation of the same promoters, suggesting a partial redundancy of their effect [96]. Nevertheless, recent data demonstrated that p53 SNP variants possess distinct transcriptional and DNA-binding properties [102]. The transactivation potential of p63 and p73 proteins also depends on their oligomeric state. Indeed, TAp63α forms an inactive, dimeric and compact conformation in resting oocytes, while the detection of DNA damage leads to the formation of an active, tetrameric and open conformation [103]. In contrast to p63, TAp73α is a constitutive open tetramer [103]. Each member of the family can act on unique targets, strongly suggesting that these proteins play not completely overlapping roles in the cell. The latter statement is further confirmed by the knock-out mice models. In particular, p53^−/−^ mice develop normally but have a very high predisposition to tumor transformation [104]; indeed, p53 somatic mutations can be found in almost two thirds of human cancers [105,106]. Skin-specific p53-null background resulted in accelerated formation of spontaneous tumors and enhanced metastasis [107]. On the other hand, p63^−/−^ mice show limb truncations, craniofacial abnormalities and die early after birth as a consequence of dehydration: these mice fail in fact to develop skin and other epithelial tissues, demonstrating the pivotal role played by p63 in epidermal morphogenesis [108–110]. In addition, loss of p63 can affect reprogramming via several mechanisms such as reduced expression of mesenchymal-epithelial transition and pluripotency genes [111]. Interestingly, p63 heterozygous mice have a shortened life-span and show features of accelerating aging [29]. As for the specific role of p63 in apoptosis, recent data highlighted the contribution of p63 pathway in damaged oocyte elimination in adulthood [112]. Finally, p73^−/−^ mice are viable but show severe neurological defects, including hydrocephalus, hippocampal dysgenesis, as well as abnormalities in pheromone sensory pathways and chronic inflammations [113,114]. Although p73 is dispensable for commitment to neural stem cell fate, it has been demonstrated to be essential for neural stem cell maintenance and for blocking premature differentiation [115–118]. Another unique role of TAp73 is in macrophage-mediated innate immunity and in the resolution of inflammatory responses [119]. In contrast to p53, both p63 and p73 are rarely mutated in cancers but they frequently display an altered ratio between TA- and ∆N-isoforms [120–122]. In the next paragraphs we will analyze the roles that p53 family plays in the cell and the connection between p53 family, DNA damage and aging [123].

### p53 family in DNA damage

Upon DNA damage induction, p53 family members are stabilized through different mechanisms. Indeed, post-translational modifications play a key role in modulating the activation, stabilization or degradation of these proteins [124]. For example, in unstressed conditions p53 is maintained at low levels by the MDM2-mediated degradation, while this regulatory mechanism is released following p53 phosphorylation by the master kinases of the DDR, such as ATM, ATR, DNA-PK and Chk2 [125–128]. In addition to prevent its degradation, these and other post-translational modifications (such as ubiquitination, acetylation or methylation) serve also to increase the transcriptional activity of p53 protein [129,130]. Finally, it should be mentioned that in its function of transcriptional regulator, p53 is directly involved in promoting the transcription of genes of the DNA damage response such as MSH2 [131,132] and FANCC [133]. With regard to p63, the DNA damage-induced phosphorylation could exert opposite roles, depending on the p63 isoforms involved. In particular, c-Abl-mediated phosphorylation can promote TAp63α switch from the inactive dimer to the active tetramer in mammalian oocytes, to protect the female germ line during meiotic arrest [134–136]. On the contrary, ∆Np63α is rapidly phosphorylated by ATM and other kinases in head and neck squamous cell carcinomas (HNSCC) upon different treatments: this modification primes ∆Np63α for degradation to promote apoptosis of the damaged cells [137]. Likewise, p73 is a target of different protein modifiers, such as the kinases c-Abl, Chk1 and Chk2 or the acetyltransferase p300 that, after DNA damage induction, activate p73 protein [138–140]. Similarly to p63, the ∆N-isoform of p73 has to be degraded to allow apoptosis induction [139,141]. Of note, it has also been demonstrated that TAp73 knockout mice show an increase of DNA damage rate in spermatogonia that finally leads to male infertility. Thus, p73 has been proposed as the guardian of male germ line [142].

### p53 family in the control of cellular senescence

Consistent with their accumulation after genotoxic stress, p53-family proteins are functionally involved in the induction of different DNA damage responses that determine cellular fate. In particular, they can promote transient cell cycle arrest to allow proper DNA repair and to prevent potentially tumorigenic cells from dividing. However, in case of continuous or irreparable DNA damage, the exit from cell cycle becomes permanent, leading to the process known as cellular senescence. Cells that have undergone senescence appear enlarged and flattened, become refractory to mitogens or growth factors and show dramatic changes in gene expression [143] and chromatin structure [144, 145]. Interestingly, progressive accumulation of senescent cells has been associated to aging *in vivo* [146]. Mechanistically, senescence is driven by two main interconnected still independent pathways that halt cell proliferation: the p53-p21 and the p16-Rb. Both p21 and p16 are cyclin-dependent kinase inhibitors (CDKI) that, among other activities, prevent phosphorylation, hence inactivation, of Rb [144, 145]. The overall effect is the suppression of E2F activity, a transcription factor that promotes the expression of genes required for cell cycle-progression [147, 148]. With reference to the involvement of p53 family members in this program, the direct induction of p21 promoter by p53 [149], TAp63 [150] or TAp73 [151] isoforms in response to genotoxic stress has been demonstrated. ROS production is well known to provoke senescence and genes that are induced by p53 may also play a relevant role in senescence induction [152]. Nevertheless, p53 also promotes the expression of several antioxidant genes which account for p53 ability to reduce the oxidative stress [153]. Likely, p53′s role in increasing and decreasing oxidative stress contributes to its dual effect on senescence [154]. The role of p53 family in ROS control is further highlighted by the recent finding that TAp63 controls the expression of GLS2 which, in turn, is important in the cellular anti-oxidant pathway [155]. As for p73, studies on TAp73-null mice demonstrated that TAp73 protects against aging by regulating mitochondrial activity and preventing ROS accumulation [156].

An additional layer of complexity in the involvement of p53 family members in the regulation of senescence was added by experimental evidence demonstrating that ΔNp63α inhibits the senescence-inducing miR-138, -181a, -181b, and -130b expression by binding directly to p63-responsive elements located in close proximity to the genomic loci of these miRNAs in primary keratinocytes [157]. Thus, suppression of miR-138, -181a, -181b, and -130b expression by ΔNp63α can be regarded as a mechanism to fine-tune the balance between cellular proliferation and senescence in epidermal proliferating cells. In addition, TAp73 induces the activation of anabolic metabolism, with enhanced pentose phosphate shunt (PPP) and nucleotide biosynthesis [158]. The metabolic effect of p73 can be interpreted as a way to counteract cellular senescence rather than to support proliferation. Furthermore, p53, p63 and p73 can work together to regulate the balance between survival, cell death, and senescence [159]. In conclusion, the pro- and anti-senescence roles of the members of p53 family depend on the presence of other regulators and more in general on the cellular context.

### p53 family in apoptosis

The most investigated biological function of p53 and its family members is doubtlessly apoptosis control [160]. Although the involvement of p53 family in apoptosis is not the focus of this review, it is worth to remind that p53 is the main hub in the control of cellular fate after damage. Nevertheless, in some contexts the pro-apoptotic function of p53 depends on the presence of other transcription factors such as c-Myc that is necessary for p53-induced apoptosis in response to DNA damage *in vivo* [161]. Among the many ways in which p53 can trigger apoptosis, one of the most relevant is through the activation of PUMA (p53-upregulated modulator of apoptosis), a BH3-only protein that induces apoptosis through the mitochondrial pathway [162–164]. In addition, p53 can induce apoptosis by promoting transcription of other pro-apoptotic proteins [165] in association with other regulators. For instance, NOXA is transcriptionally induced by binding of p53 in complex with p18/Hamlet to its promoter [166]. Intriguingly, not all p53 targets are downstream executors of apoptosis. Slug (SNAI2), a central regulator of the epithelial mesenchymal transition (EMT) process that is important for tumor metastasis, is a transcriptional target of p53 but, in turn it is a repressor of PUMA thus preventing the pro-apoptotic activity of the latter gene [167]. Finally, it should be mentioned that p53-independent apoptotic pathways exist in normal and cancer cells [168]. The role of p63 and p73 in apoptosis was, at least at the beginning, largely interpreted as a functional overlapping of the pro-apoptotic function of p53. Nevertheless, there are cellular contexts in which the activity of p63 and p73 is required for p53-dependent apoptosis. Thus, p63 and p73 are required for p53-induced apoptosis after DNA damage. In fact, it has been demonstrated that the combined loss of p63 and p73 results in the failure of cells containing functional p53 to undergo apoptosis in response to DNA damage [169]. In addition, the distinct role of the p53 paralogs is evident in the case of male gonocytes where p53 is not expressed. The lack of p63 in gonocytes sharply decreases the rate of physiological apoptosis causing abnormal morphology of the germ cells [170]. Enhanced resistance to chemotherapy has been correlated with high levels of ΔNp73 [171]. Recent data demonstrated that acetylpolyamine oxidase (PAOX) upregulates ΔNp73 levels by suppressing its degradation [171]. The deficiency of the TAp73 isoform results in male infertility because of severe impairment of spermatogenesis. Accordingly, mice lacking TAp73 exhibited increased DNA damage and cell death in spermatogonia, disorganized apical ectoplasmic specialization, malformed spermatids, and marked hyperspermia [142]. Furthermore, the pro-apoptotic activity of p53 paralogs can be regulated by cross-talk with other factors. p73 induces apoptosis in T cells by promoting the transcription of Bim. In activated T cells NF-κB induces Mdm2 that in turn, forms a complex with p73 thus inhibiting p73–dependent activation of Bim and the resulting apoptosis [172]. Noteworthy, although apoptosis is the most common form of cell demise induced by p53 family members, there are cases in which p53 promotes other types of cellular death [173].

### p53 family in the regulation of the aging process

A less obvious, nonetheless relevant function of the p53 family members is in cellular aging through their activity on DNA repair mechanisms. p53, as well as its paralogs, can influence the aging process by regulating core DNA repair proteins expression, by directly interacting with repair factors or by inducing apoptosis in response to DNA damage. A paradigmatic example of the link p53-DNA repair-aging is the role of p53 in the progeroid Cockayne syndrome [174]. Complementation group B (CSB) protein is an ATP-dependent chromatin remodeler with an essential function in transcription-coupled DNA repair. Mutations in the CSB gene are frequent in Cockayne syndrome. Normally, p53 tumor protein interacts with CSB, and the chromatin association of CSB and p53 is inversely related. CSB facilitates the sequence-independent association of p53 with chromatin when p53 concentrations are low and p53 prevents CSB from binding to nucleosomes when p53 concentrations are elevated. These results suggest that the reciprocal regulation of chromatin access by CSB and p53 could be part of a mechanism by which these two proteins coordinate their activities to regulate DNA repair, cell survival and aging.

Genotoxic stresses such as UV irradiation and exposure to environmental pollutants cause damage to cellular DNA, a powerful driver of senescence and aging. Besides acting directly in the control of genes involved in the UV-induced DNA damage response, p53 promotes the transcription of genes that block the cell cycle progression (i.e. p21) and regulates the expression of different cyclin-dependent kinase inhibitors (CDKIs) thus contributing to the proliferative stop necessary to allow the execution of DNA repair [175]. In this context, the activity of p53 can be regarded as a shielding mechanism to maintain the genome stability necessary to prevent the aging process [175].

The mechanisms that drive neuronal impairment in the central nervous system (CNS) are of paramount importance to understand the functional decline that occurs in aging brains. In an animal model that utilized hydroxyurea (HU) treatment, the phenotypic features of cellular senescence [176] such as senescence-associated-β-galactosidase (SA-β-gal) staining, decreased proliferation and differentiation capacity, increased G0/G1 cell cycle arrest, elevated ROS level and diminished apoptosis, were accompanied by a marked increase of p53 expression, as well as a decreased expression of key proteins in various DNA repair pathways such as XRCC2, XRCC3 and Ku70 [177]. Here, p53 activity is necessary for G1/S cell cycle arrest and to mitigate the devastating consequences induced by DNA damage and allow time to repair. Nevertheless, p53-mediated G1 arrest can alternatively coax cells to enter a senescence state that precedes aging. In this system, p53 acts as a modulator of the biological response by directing cells to DNA repair or towards aging depending on the extent of the damage.

As for the p53 paralogs, it is well known that they have distinctive functions in the DNA damage response. In fact, TAp73 and TAp63 (but not p53) promote the transcription of BRCA1, RAD50 and MRE11 which are involved in HR [93]. Nevertheless, their link to the aging process has just begun to be investigated in detail. The first experimental evidence of a role of TAp73 in aging comes from an in vivo study in which it has been demonstrated that TAp73-null mice show more pronounced aging with increased oxidative damage and senescence due to metabolic dysregulation [156]. ∆Np73 localizes directly to the site of DNA damage, can interact with the DNA damage sensor protein 53BP1, and inhibits ATM activation and subsequent p53 phosphorylation [178]. This finding is particularly interesting in light of the recent discovery of a considerable number of transcription factors localized in the vicinity of the sites of DNA damage [95]. In addition, E1A- and RASV12-transformed ∆Np73^−/−^ MEFs display a significant delay in tumor initiation, as well as a decrease in tumor size compared with wild-type controls [179]. Intriguingly, the reduced tumorigenic ability of ∆Np73^−/−^ MEFs to form tumors was not due to a higher level of apoptosis, but instead to enhanced cellular senescence, as demonstrated by increased expression of the senescence markers p16Ink4a, senescence-associated β-galactosidase (SA-β-gal), and DcR2 [179]. Since ∆Np73^−/−^ mice show signs of neurodegeneration in their brain [178], it is feasible that the lack of ∆Np73 contributes to a process of senescence (and subsequent aging) in neuronal tissues. In line with an anti-aging role of p73, it has been demonstrated that TAp73 is a major regulator of autophagy by promoting the expression of ATG5 thus preventing aging by maintaining a homeostatic control [180].

The development of inducible TAp63-KO mice has contributed to the understanding of the role of p63 in senescence and aging. In the epithelial compartment, the lack of p63 accelerates aging as indicated by the accumulation of senescence markers [29]. Furthermore, TAp63 isoform specific KO highlighted skin ulceration, premature aging and reduced lifespan that are associated with genomic instability [181, 182] which may be due to a still incompletely defined role of p63 in DNA repair processes. Although the above reported results highlighted an anti-aging function of p63, it should be mentioned that TAp63 mediates the induction of oncogene induced senescence (OIS) in keratinocytes [183]. Thus it is likely that similarly to p53, p63 can promote senescence upon oncogenic stress but, in normal conditions, its absence accelerates aging. A complete understanding of the role of p63 in the aging process is furthermore complicated by the existence of N-terminal truncated isoforms (∆Np63) [184] that can have distinct functions.

### Concluding remarks

**Figure 4:**
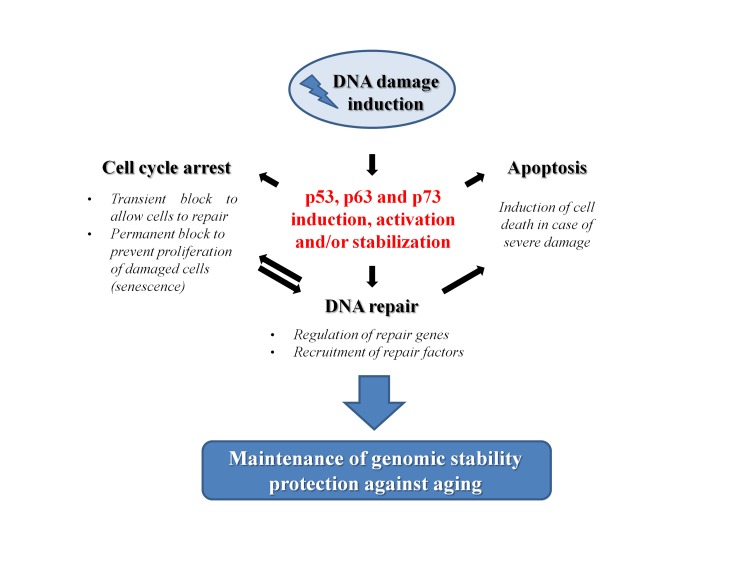
The role of p53 family members in the prevention of aging p53 family proteins are involved in different mechanisms after the induction of a DNA damage. A transient block of cell cycle progression gives time to the cells to repair the DNA. Nevertheless, p53 family proteins can also directly affect the DNA repair mechanisms (see text for details). However, if the damage is too extensive or cannot be repaired, p53 family members could trigger either a permanent exit from the cell cycle (senescence) or a cell death program (apoptosis) as mechanisms of defense. The overall result can be read as a check of the genomic stability that protects cells from aging.

Genomic instability is tightly linked to the aging process. As the integrity of DNA is continuously challenged by endogenous and exogenous factors, the mechanisms that safeguard genome maintenance can be considered to all intents and purposes as anti-aging processes. Among these, the different and specific repair mechanisms that cells have developed to restore the chemical structure of the DNA molecule should be unquestionably included. However, in addition to the core proteins, that are instrumental for the execution of the repair processes, there is an ever growing number of accessory proteins involved in these mechanisms that indirectly help the DNA repair. In particular, transcription factors can support DNA repair by regulating the expression of key factors of the DNA repair machinery. Nevertheless, they can also recruit different chromatin remodeling complexes to the DNA damage sites, thus mediating the accessibility of the DNA that has to be repaired. Furthermore, they are also usually involved in the regulation of the other processes strictly linked to the repair mechanisms that occur during the DNA damage response. In this context, the p53 family plays multiple important roles in modulating the aging process (Figure 4). Following a DNA damage induction, the members of this family are induced or stabilized, usually through post-translational modifications of the proteins. In their active form, they can exert partially overlapped roles in regulating processes that determine the fate of the damaged cells. Depending on the extent of the damage, their role can vary from (i) the induction of a transient cell cycle arrest to allow DNA repair, to (ii) the induction of a permanent blockade of proliferation resulting in senescence, to (iii) the stimulation of apoptosis to eliminate damaged cells. In their quality of transcriptional regulators p53, p63 and p73 can also influence the efficiency of repair mechanisms by regulating the expression of key factors or by directly interacting with them as we saw in the previous paragraphs. In light of these evidences, there is no doubt that the p53 family plays a central role in maintaining the genome stability. As a direct consequence of this, we can conclude that their function strongly impacts on the complex regulation of the aging process.

## References

[1] Kirkwood TB (2005). Understanding the odd science of aging. Cell.

[2] Wilson DM, Bohr VA, McKinnon PJ (2008). DNA damage, DNA repair, ageing and age-related disease. Mech Ageing Dev.

[3] Gems D, Partridge L (2013). Genetics of longevity in model organisms: debates and paradigm shifts. Annu Rev Physiol.

[4] Vijg J, Campisi J (2008). Puzzles, promises and a cure for ageing. Nature.

[5] Lopez-Otin C, Blasco MA, Partridge L, Serrano M, Kroemer G (2013). The hallmarks of aging. Cell.

[6] Moskalev AA, Shaposhnikov MV, Plyusnina EN, Zhavoronkov A, Budovsky A, Yanai H (2013). The role of DNA damage and repair in aging through the prism of Koch-like criteria. Ageing Res Rev.

[7] Hughes KA, Reynolds RM (2005). Evolutionary and mechanistic theories of aging. Annu Rev Entomol.

[8] Lindahl T (1993). Instability and decay of the primary structure of DNA. Nature.

[9] Martin GM (2007). Modalities of gene action predicted by the classical evolutionary biological theory of aging. Ann N Y Acad Sci.

[10] Peuget S, Bonacci T, Soubeyran P, Iovanna J, Dusetti NJ (2014). Oxidative stress-induced p53 activity is enhanced by a redox-sensitive TP53INP1 SUMOylation. Cell Death Differ.

[11] Zhang HH, Li SZ, Zhang ZY, Hu XM, Hou PN, Gao L (2014). Nemo-like kinase is critical for p53 stabilization and function in response to DNA damage. Cell Death Differ.

[12] Finkel T, Holbrook NJ (2000). Oxidants, oxidative stress and the biology of ageing. Nature.

[13] Hoeijmakers JH (2009). DNA damage, aging, and cancer. N Engl J Med.

[14] Sohal RS, Weindruch R (1996). Oxidative stress, caloric restriction, and aging. Science.

[15] Shi Y, Nikulenkov F, Zawacka-Pankau J, Li H, Gabdoulline R, Xu J (2014). ROS-dependent activation of JNK converts p53 into an efficient inhibitor of oncogenes leading to robust apoptosis. Cell Death Differ.

[16] Marnett LJ, Plastaras JP (2001). Endogenous DNA damage and mutation. Trends Genet.

[17] Sedelnikova OA, Horikawa I, Zimonjic DB, Popescu NC, Bonner WM, Barrett JC (2004). Senescing human cells and ageing mice accumulate DNA lesions with unrepairable double-strand breaks. Nat Cell Biol.

[18] Harman D (2003). The free radical theory of aging. Antioxid Redox Signal.

[19] Little JB (1976). Relationship between DNA repair capacity and cellular aging. Gerontology.

[20] Lombard DB, Chua KF, Mostoslavsky R, Franco S, Gostissa M, Alt FW (2005). DNA repair, genome stability, and aging. Cell.

[21] Lou Z, Chen J (2006). Cellular senescence and DNA repair. Exp Cell Res.

[22] Kook SY, Jeong H, Kang MJ, Park R, Shin HJ, Han SH (2014). Crucial role of calbindin-D28k in the pathogenesis of Alzheimer's disease mouse model. Cell Death Differ.

[23] Martin GM, Oshima J (2000). Lessons from human progeroid syndromes. Nature.

[24] Navarro CL, Cau P, Levy N (2006). Molecular bases of progeroid syndromes. Hum Mol Genet.

[25] Burnley P, Rahman M, Wang H, Zhang Z, Sun X, Zhuge Q (2013). Role of the p63-FoxN1 regulatory axis in thymic epithelial cell homeostasis during aging. Cell Death Dis.

[26] Cao L, Li W, Kim S, Brodie SG, Deng CX (2003). Senescence, aging, and malignant transformation mediated by p53 in mice lacking the Brca1 full-length isoform. Genes Dev.

[27] Feng Z, Hu W, Teresky AK, Hernando E, Cordon-Cardo C, Levine AJ (2007). Declining p53 function in the aging process: a possible mechanism for the increased tumor incidence in older populations. Proc Natl Acad Sci U S A.

[28] Gambino V, De Michele G, Venezia O, Migliaccio P, Dall'Olio V, Bernard L (2013). Oxidative stress activates a specific p53 transcriptional response that regulates cellular senescence and aging. Aging Cell.

[29] Keyes WM, Wu Y, Vogel H, Guo X, Lowe SW, Mills AA (2005). p63 deficiency activates a program of cellular senescence and leads to accelerated aging. Genes Dev.

[30] Kim J, Nakasaki M, Todorova D, Lake B, Yuan CY, Jamora C (2014). p53 induces skin aging by depleting Blimp1+ sebaceous gland cells. Cell Death Dis.

[31] Rufini A, Tucci P, Celardo I, Melino G (2013). Senescence and aging: the critical roles of p53. Oncogene.

[32] Wetzel MK, Naska S, Laliberte CL, Rymar VV, Fujitani M, Biernaskie JA (2008). p73 regulates neurodegeneration and phospho-tau accumulation during aging and Alzheimer's disease. Neuron.

[33] Cong L, Ran FA, Cox D, Lin S, Barretto R, Habib N (2013). Multiplex genome engineering using CRISPR/Cas systems. Science.

[34] Mali P, Yang L, Esvelt KM, Aach J, Guell M, DiCarlo JE (2013). RNA-guided human genome engineering via Cas9. Science.

[35] Harper JW, Elledge SJ (2007). The DNA damage response: ten years after. Mol Cell.

[36] Ogrunc M, Di MR, Liontos M, Bombardelli L, Mione M, Fumagalli M (2014). Oncogene-induced reactive oxygen species fuel hyperproliferation and DNA damage response activation. Cell Death Differ.

[37] Shetzer Y, Kagan S, Koifman G, Sarig R, Kogan-Sakin I, Charni M (2014). The onset of p53 loss of heterozygosity is differentially induced in various stem cell types and may involve the loss of either allele. Cell Death Differ.

[38] Zhou BB, Elledge SJ (2000). The DNA damage response: putting checkpoints in perspective. Nature.

[39] Tilgner K, Neganova I, Moreno-Gimeno I, Al-Aama JY, Burks D, Yung S (2013). A human iPSC model of Ligase IV deficiency reveals an important role for NHEJ-mediated-DSB repair in the survival and genomic stability of induced pluripotent stem cells and emerging haematopoietic progenitors. Cell Death Differ.

[40] Campisi J (2005). Aging, tumor suppression and cancer: high wire-act!. Mech Ageing Dev.

[41] Hoeijmakers JH (2001). Genome maintenance mechanisms for preventing cancer. Nature.

[42] Almeida KH, Sobol RW (2007). A unified view of base excision repair: lesion-dependent protein complexes regulated by post-translational modification. DNA Repair (Amst).

[43] Wilson DM, Bohr VA (2007). The mechanics of base excision repair, and its relationship to aging and disease. DNA Repair (Amst).

[44] David SS, O'Shea VL, Kundu S (2007). Base-excision repair of oxidative DNA damage. Nature.

[45] Hasty P, Campisi J, Hoeijmakers J, van SH, Vijg J (2003). Aging and genome maintenance: lessons from the mouse?. Science.

[46] Tebbs RS, Flannery ML, Meneses JJ, Hartmann A, Tucker JD, Thompson LH (1999). Requirement for the Xrcc1 DNA base excision repair gene during early mouse development. Dev Biol.

[47] Atamna H, Cheung I, Ames BN (2000). A method for detecting abasic sites in living cells: age-dependent changes in base excision repair. Proc Natl Acad Sci U S A.

[48] Gorbunova V, Seluanov A, Mao Z, Hine C (2007). Changes in DNA repair during aging. Nucleic Acids Res.

[49] Hamilton ML, Van RH, Drake JA, Yang H, Guo ZM, Kewitt K (2001). Does oxidative damage to DNA increase with age?. Proc Natl Acad Sci U S A.

[50] Imam SZ, Karahalil B, Hogue BA, Souza-Pinto NC, Bohr VA (2006). Mitochondrial and nuclear DNA-repair capacity of various brain regions in mouse is altered in an age-dependent manner. Neurobiol Aging.

[51] Lans H, Lindvall JM, Thijssen K, Karambelas AE, Cupac D, Fensgard O (2013). DNA damage leads to progressive replicative decline but extends the life span of long-lived mutant animals. Cell Death Differ.

[52] Fousteri M, Mullenders LH (2008). Transcription-coupled nucleotide excision repair in mammalian cells: molecular mechanisms and biological effects. Cell Res.

[53] Gillet LC, Scharer OD (2006). Molecular mechanisms of mammalian global genome nucleotide excision repair. Chem Rev.

[54] Hanawalt PC (2002). Subpathways of nucleotide excision repair and their regulation. Oncogene.

[55] Sugasawa K (2006). UV-induced ubiquitylation of XPC complex, the UV-DDB-ubiquitin ligase complex, and DNA repair. J Mol Histol.

[56] de Laat WL, Jaspers NG, Hoeijmakers JH (1999). Molecular mechanism of nucleotide excision repair. Genes Dev.

[57] Kraemer KH, Patronas NJ, Schiffmann R, Brooks BP, Tamura D, DiGiovanna JJ (2007). Xeroderma pigmentosum, trichothiodystrophy and Cockayne syndrome: a complex genotype-phenotype relationship. Neuroscience.

[58] de Boer J, Andressoo JO, de WJ, Huijmans J, Beems RB, van SH (2002). Premature aging in mice deficient in DNA repair and transcription. Science.

[59] Gorbunova V, Seluanov A, Mao Z, Hine C (2007). Changes in DNA repair during aging. Nucleic Acids Res.

[60] Mitchell JR, Hoeijmakers JH, Niedernhofer LJ (2003). Divide and conquer: nucleotide excision repair battles cancer and ageing. Curr Opin Cell Biol.

[61] Bassing CH, Alt FW (2004). The cellular response to general and programmed DNA double strand breaks. DNA Repair (Amst).

[62] Jackson SP (2002). Sensing and repairing DNA double-strand breaks. Carcinogenesis.

[63] Salomoni P (2013). Reprogramming and genome integrity: role of non-homologous end joining. Cell Death Differ.

[64] Vyas R, Kumar R, Clermont F, Helfricht A, Kalev P, Sotiropoulou P (2013). RNF4 is required for DNA double-strand break repair in vivo. Cell Death Differ.

[65] Bassing CH, Alt FW (2004). The cellular response to general and programmed DNA double strand breaks. DNA Repair (Amst).

[66] Moynahan ME, Jasin M (2010). Mitotic homologous recombination maintains genomic stability and suppresses tumorigenesis. Nat Rev Mol Cell Biol.

[67] Wyman C, Ristic D, Kanaar R (2004). Homologous recombination-mediated double-strand break repair. DNA Repair (Amst).

[68] Titus S, Li F, Stobezki R, Akula K, Unsal E, Jeong K (2013). Impairment of BRCA1-related DNA double-strand break repair leads to ovarian aging in mice and humans. Sci Transl Med.

[69] Couzin-Frankel J (2013). Reproductive Biology. Faulty DNA repair linked to ovarian aging in mice and humans. Science.

[70] Sonoda E, Hochegger H, Saberi A, Taniguchi Y, Takeda S (2006). Differential usage of non-homologous end-joining and homologous recombination in double strand break repair. DNA Repair (Amst).

[71] Lieber MR (2010). NHEJ and its backup pathways in chromosomal translocations. Nat Struct Mol Biol.

[72] O'Driscoll M, Jeggo PA (2006). The role of double-strand break repair - insights from human genetics. Nat Rev Genet.

[73] Karanjawala ZE, Lieber MR (2004). DNA damage and aging. Mech Ageing Dev.

[74] Wang B, Xie M, Li R, Owonikoko TK, Ramalingam SS, Khuri FR (2014). Role of Ku70 in deubiquitination of Mcl-1 and suppression of apoptosis. Cell Death Differ.

[75] Lieber MR (2008). The mechanism of human nonhomologous DNA end joining. J Biol Chem.

[76] Waters CA, Strande NT, Wyatt DW, Pryor JM, Ramsden DA (2014). Nonhomologous end joining: a good solution for bad ends. DNA Repair (Amst).

[77] Woodbine L, Gennery AR, Jeggo PA (2014). The clinical impact of deficiency in DNA non-homologous end-joining. DNA Repair (Amst).

[78] Craxton A, Somers J, Munnur D, Jukes-Jones R, Cain K, Malewicz M (2015). XLS (c9orf142) is a new component of mammalian DNA double-stranded break repair. Cell Death Differ.

[79] Ochi T, Blackford AN, Coates J, Jhujh S, Mehmood S, Tamura N (2015). DNA repair. PAXX, a paralog of XRCC4 and XLF, interacts with Ku to promote DNA double-strand break repair. Science.

[80] Xing M, Yang M, Huo W, Feng F, Wei L, Jiang W (2015). Interactome analysis identifies a new paralogue of XRCC4 in non-homologous end joining DNA repair pathway. Nat Commun.

[81] Seluanov A, Mittelman D, Pereira-Smith OM, Wilson JH, Gorbunova V (2004). DNA end joining becomes less efficient and more error-prone during cellular senescence. Proc Natl Acad Sci U S A.

[82] Seluanov A, Danek J, Hause N, Gorbunova V (2007). Changes in the level and distribution of Ku proteins during cellular senescence. DNA Repair (Amst).

[83] Difilippantonio MJ, Zhu J, Chen HT, Meffre E, Nussenzweig MC, Max EE (2000). DNA repair protein Ku80 suppresses chromosomal aberrations and malignant transformation. Nature.

[84] Frank KM, Sharpless NE, Gao Y, Sekiguchi JM, Ferguson DO, Zhu C (2000). DNA ligase IV deficiency in mice leads to defective neurogenesis and embryonic lethality via the p53 pathway. Mol Cell.

[85] Gao Y, Ferguson DO, Xie W, Manis JP, Sekiguchi J, Frank KM (2000). Interplay of p53 and DNA-repair protein XRCC4 in tumorigenesis, genomic stability and development. Nature.

[86] Ferguson DO, Alt FW (2001). DNA double strand break repair and chromosomal translocation: lessons from animal models. Oncogene.

[87] Nussenzweig A (2007). Causes and consequences of the DNA damage response. Cell Cycle.

[88] McVey M, Lee SE (2008). MMEJ repair of double-strand breaks (director's cut): deleted sequences and alternative endings. Trends Genet.

[89] Malewicz M, Kadkhodaei B, Kee N, Volakakis N, Hellman U, Viktorsson K (2011). Essential role for DNA-PK-mediated phosphorylation of NR4A nuclear orphan receptors in DNA double-strand break repair. Genes Dev.

[90] Venere M, Hamerlik P, Wu Q, Rasmussen RD, Song LA, Vasanji A (2014). Therapeutic targeting of constitutive PARP activation compromises stem cell phenotype and survival of glioblastoma-initiating cells. Cell Death Differ.

[91] Rulten SL, Fisher AE, Robert I, Zuma MC, Rouleau M, Ju L (2011). PARP-3 and APLF function together to accelerate nonhomologous end-joining. Mol Cell.

[92] Goldstein M, Derheimer FA, Tait-Mulder J, Kastan MB (2013). Nucleolin mediates nucleosome disruption critical for DNA double-strand break repair. Proc Natl Acad Sci U S A.

[93] Lin YL, Sengupta S, Gurdziel K, Bell GW, Jacks T, Flores ER (2009). p63 and p73 transcriptionally regulate genes involved in DNA repair. PLoS Genet.

[94] Pieraccioli M, Nicolai S, Antonov A, Somers J, Malewicz M, Melino G (2015). ZNF281 contributes to the DNA damage response by controlling the expression of XRCC2 and XRCC4. Oncogene.

[95] Izhar L, Adamson B, Ciccia A, Lewis J, Pontano-Vaites L, Leng Y (2015). A Systematic Analysis of Factors Localized to Damaged Chromatin Reveals PARP-Dependent Recruitment of Transcription Factors. Cell Rep.

[96] Murray-Zmijewski F, Lane DP, Bourdon JC (2006). p53/p63/p73 isoforms: an orchestra of isoforms to harmonise cell differentiation and response to stress. Cell Death Differ.

[97] Vanbokhoven H, Melino G, Candi E, Declercq W (2011). p63, a story of mice and men. J Invest Dermatol.

[98] Bourdon JC, Fernandes K, Murray-Zmijewski F, Liu G, Diot A, Xirodimas DP (2005). p53 isoforms can regulate p53 transcriptional activity. Genes Dev.

[99] Chen J, Ng SM, Chang C, Zhang Z, Bourdon JC, Lane DP (2009). p53 isoform delta113p53 is a p53 target gene that antagonizes p53 apoptotic activity via BclxL activation in zebrafish. Genes Dev.

[100] De Laurenzi V, Costanzo A, Barcaroli D, Terrinoni A, Falco M, Annicchiarico-Petruzzelli M (1998). Two new p73 splice variants, gamma and delta, with different transcriptional activity. J Exp Med.

[101] Marcel V, Fernandes K, Terrier O, Lane DP, Bourdon JC (2014). Modulation of p53beta and p53gamma expression by regulating the alternative splicing of TP53 gene modifies cellular response. Cell Death Differ.

[102] Wang B, Niu D, Lam TH, Xiao Z, Ren EC (2014). Mapping the p53 transcriptome universe using p53 natural polymorphs. Cell Death Differ.

[103] Luh LM, Kehrloesser S, Deutsch GB, Gebel J, Coutandin D, Schafer B (2013). Analysis of the oligomeric state and transactivation potential of TAp73alpha. Cell Death Differ.

[104] Donehower LA, Harvey M, Slagle BL, McArthur MJ, Montgomery CA, Butel JS (1992). Mice deficient for p53 are developmentally normal but susceptible to spontaneous tumours. Nature.

[105] Olivier M, Hollstein M, Hainaut P (2010). TP53 mutations in human cancers: origins, consequences, and clinical use. Cold Spring Harb Perspect Biol.

[106] Vousden KH, Lu X (2002). Live or let die: the cell's response to p53. Nat Rev Cancer.

[107] De Craene B, Denecker G, Vermassen P, Taminau J, Mauch C, Derore A (2014). Epidermal Snail expression drives skin cancer initiation and progression through enhanced cytoprotection, epidermal stem/progenitor cell expansion and enhanced metastatic potential. Cell Death Differ.

[108] Mills AA, Zheng B, Wang XJ, Vogel H, Roop DR, Bradley A (1999). p63 is a p53 homologue required for limb and epidermal morphogenesis. Nature.

[109] Yallowitz AR, Alexandrova EM, Talos F, Xu S, Marchenko ND, Moll UM (2014). p63 is a prosurvival factor in the adult mammary gland during post-lactational involution, affecting PI-MECs and ErbB2 tumorigenesis. Cell Death Differ.

[110] Yang A, Schweitzer R, Sun D, Kaghad M, Walker N, Bronson RT (1999). p63 is essential for regenerative proliferation in limb, craniofacial and epithelial development. Nature.

[111] Alexandrova EM, Petrenko O, Nemajerova A, Romano RA, Sinha S, Moll UM (2013). DeltaNp63 regulates select routes of reprogramming via multiple mechanisms. Cell Death Differ.

[112] Vandormael-Pournin S, Guigon CJ, Ishaq M, Coudouel N, Ave P, Huerre M (2015). Oocyte-specific inactivation of Omcg1 leads to DNA damage and c-Abl/TAp63-dependent oocyte death associated with dramatic remodeling of ovarian somatic cells. Cell Death Differ.

[113] Pozniak CD, Barnabe-Heider F, Rymar VV, Lee AF, Sadikot AF, Miller FD (2002). p73 is required for survival and maintenance of CNS neurons. J Neurosci.

[114] Yang A, Walker N, Bronson R, Kaghad M, Oosterwegel M, Bonnin J (2000). p73-deficient mice have neurological, pheromonal and inflammatory defects but lack spontaneous tumours. Nature.

[115] Alexandrova EM, Talos F, Moll UM (2013). p73 is dispensable for commitment to neural stem cell fate, but is essential for neural stem cell maintenance and for blocking premature differentiation. Cell Death Differ.

[116] Agostini M, Tucci P, Chen H, Knight RA, Bano D, Nicotera P (2010). p73 regulates maintenance of neural stem cell. Biochem Biophys Res Commun.

[117] Fujitani M, Cancino GI, Dugani CB, Weaver IC, Gauthier-Fisher A, Paquin A (2010). TAp73 acts via the bHLH Hey2 to promote long-term maintenance of neural precursors. Curr Biol.

[118] Gonzalez-Cano L, Herreros-Villanueva M, Fernandez-Alonso R, Ayuso-Sacido A, Meyer G, Garcia-Verdugo JM (2010). p73 deficiency results in impaired self renewal and premature neuronal differentiation of mouse neural progenitors independently of p53. Cell Death Dis.

[119] Tomasini R, Secq V, Pouyet L, Thakur AK, Wilhelm M, Nigri J (2013). TAp73 is required for macrophage-mediated innate immunity and the resolution of inflammatory responses. Cell Death Differ.

[120] Candi E, Dinsdale D, Rufini A, Salomoni P, Knight RA, Mueller M (2007). TAp63 and DeltaNp63 in cancer and epidermal development. Cell Cycle.

[121] Melino G, De L V, Vousden KH (2002). p73: Friend or foe in tumorigenesis. Nat Rev Cancer.

[122] Moll UM, Slade N (2004). p63 and p73: roles in development and tumor formation. Mol Cancer Res.

[123] Adamovich Y, Adler J, Meltser V, Reuven N, Shaul Y (2014). AMPK couples p73 with p53 in cell fate decision. Cell Death Differ.

[124] Liu J, Zhang C, Wang XL, Ly P, Belyi V, Xu-Monette ZY (2014). E3 ubiquitin ligase TRIM32 negatively regulates tumor suppressor p53 to promote tumorigenesis. Cell Death Differ.

[125] Bohgaki M, Hakem A, Halaby MJ, Bohgaki T, Li Q, Bissey PA (2013). The E3 ligase PIRH2 polyubiquitylates CHK2 and regulates its turnover. Cell Death Differ.

[126] Nair BC, Krishnan SR, Sareddy GR, Mann M, Xu B, Natarajan M (2014). Proline, glutamic acid and leucine-rich protein-1 is essential for optimal p53-mediated DNA damage response. Cell Death Differ.

[127] Serrano MA, Li Z, Dangeti M, Musich PR, Patrick S, Roginskaya M (2013). DNA-PK, ATM and ATR collaboratively regulate p53-RPA interaction to facilitate homologous recombination DNA repair. Oncogene.

[128] Taylor WR, Stark GR (2001). Regulation of the G2/M transition by p53. Oncogene.

[129] Appella E, Anderson CW (2001). Post-translational modifications and activation of p53 by genotoxic stresses. Eur J Biochem.

[130] Meek DW (2004). The p53 response to DNA damage. DNA Repair (Amst).

[131] Scherer SJ, Welter C, Zang KD, Dooley S (1996). Specific in vitro binding of p53 to the promoter region of the human mismatch repair gene hMSH2. Biochem Biophys Res Commun.

[132] Toft NJ, Winton DJ, Kelly J, Howard LA, Dekker M, te RH (1999). Msh2 status modulates both apoptosis and mutation frequency in the murine small intestine. Proc Natl Acad Sci U S A.

[133] Liebetrau W, Budde A, Savoia A, Grummt F, Hoehn H (1997). p53 activates Fanconi anemia group C gene expression. Hum Mol Genet.

[134] Amelio I, Grespi F, Annicchiarico-Petruzzelli M, Melino G (2012). p63 the guardian of human reproduction. Cell Cycle.

[135] Deutsch GB, Zielonka EM, Coutandin D, Weber TA, Schafer B, Hannewald J (2011). DNA damage in oocytes induces a switch of the quality control factor TAp63alpha from dimer to tetramer. Cell.

[136] Suh EK, Yang A, Kettenbach A, Bamberger C, Michaelis AH, Zhu Z (2006). p63 protects the female germ line during meiotic arrest. Nature.

[137] Huang Y, Sen T, Nagpal J, Upadhyay S, Trink B, Ratovitski E (2008). ATM kinase is a master switch for the Delta Np63 alpha phosphorylation/degradation in human head and neck squamous cell carcinoma cells upon DNA damage. Cell Cycle.

[138] Costanzo A, Merlo P, Pediconi N, Fulco M, Sartorelli V, Cole PA (2002). DNA damage-dependent acetylation of p73 dictates the selective activation of apoptotic target genes. Mol Cell.

[139] Maisse C, Munarriz E, Barcaroli D, Melino G, De Laurenzi V (2004). DNA damage induces the rapid and selective degradation of the DeltaNp73 isoform, allowing apoptosis to occur. Cell Death Differ.

[140] Urist M, Tanaka T, Poyurovsky MV, Prives C (2004). p73 induction after DNA damage is regulated by checkpoint kinases Chk1 and Chk2. Genes Dev.

[141] Vernersson-Lindahl E, Mills AA (2010). {Delta}Np73{beta} puts the brakes on DNA repair. Genes Dev.

[142] Inoue S, Tomasini R, Rufini A, Elia AJ, Agostini M, Amelio I (2014). TAp73 is required for spermatogenesis and the maintenance of male fertility. Proc Natl Acad Sci U S A.

[143] Grasso D, Garcia MN, Hamidi T, Cano C, Calvo E, Lomberk G (2014). Genetic inactivation of the pancreatitis-inducible gene Nupr1 impairs PanIN formation by modulating Kras(G12D)-induced senescence. Cell Death Differ.

[144] Ben-Porath I, Weinberg RA (2005). The signals and pathways activating cellular senescence. Int J Biochem Cell Biol.

[145] Kuilman T, Michaloglou C, Mooi WJ, Peeper DS (2010). The essence of senescence. Genes Dev.

[146] Campisi J (2001). From cells to organisms: can we learn about aging from cells in culture?. Exp Gerontol.

[147] Campisi J, d'Adda di FF (2007). Cellular senescence: when bad things happen to good cells. Nat Rev Mol Cell Biol.

[148] Sherr CJ, McCormick F (2002). The RB and p53 pathways in cancer. Cancer Cell.

[149] Bunz F, Dutriaux A, Lengauer C, Waldman T, Zhou S, Brown JP (1998). Requirement for p53 and p21 to sustain G2 arrest after DNA damage. Science.

[150] Guo X, Keyes WM, Papazoglu C, Zuber J, Li W, Lowe SW (2009). TAp63 induces senescence and suppresses tumorigenesis in vivo. Nat Cell Biol.

[151] Vayssade M, Haddada H, Faridoni-Laurens L, Tourpin S, Valent A, Benard J (2005). P73 functionally replaces p53 in Adriamycin-treated, p53-deficient breast cancer cells. Int J Cancer.

[152] Lu T, Finkel T (2008). Free radicals and senescence. Exp Cell Res.

[153] Olovnikov IA, Kravchenko JE, Chumakov PM (2009). Homeostatic functions of the p53 tumor suppressor: regulation of energy metabolism and antioxidant defense. Semin Cancer Biol.

[154] Vigneron A, Vousden KH (2010). p53, ROS and senescence in the control of aging. Aging (Albany NY).

[155] Giacobbe A, Bongiorno-Borbone L, Bernassola F, Terrinoni A, Markert EK, Levine AJ (2013). p63 regulates glutaminase 2 expression. Cell Cycle.

[156] Rufini A, Niklison-Chirou MV, Inoue S, Tomasini R, Harris IS, Marino A (2012). TAp73 depletion accelerates aging through metabolic dysregulation. Genes Dev.

[157] Rivetti di Valcervo, Lena AM, Nicoloso M, Rossi S, Mancini M, Zhou H (2012). p63-microRNA feedback in keratinocyte senescence. Proc Natl Acad Sci U S A.

[158] Agostini M, Niklison-Chirou MV, Catani MV, Knight RA, Melino G, Rufini A (2014). TAp73 promotes anti-senescence-anabolism not proliferation. Aging (Albany NY).

[159] Fatt MP, Cancino GI, Miller FD, Kaplan DR (2014). p63 and p73 coordinate p53 function to determine the balance between survival, cell death, and senescence in adult neural precursor cells. Cell Death Differ.

[160] Li H, Zhang Y, Strose A, Tedesco D, Gurova K, Selivanova G (2014). Integrated high-throughput analysis identifies Sp1 as a crucial determinant of p53-mediated apoptosis. Cell Death Differ.

[161] Phesse TJ, Myant KB, Cole AM, Ridgway RA, Pearson H, Muncan V (2014). Endogenous c-Myc is essential for p53-induced apoptosis in response to DNA damage in vivo. Cell Death Differ.

[162] Westphal D, Kluck RM, Dewson G (2014). Building blocks of the apoptotic pore: how Bax and Bak are activated and oligomerize during apoptosis. Cell Death Differ.

[163] Yu J, Zhang L (2003). No PUMA, no death: implications for p53-dependent apoptosis. Cancer Cell.

[164] Yu J, Wang Z, Kinzler KW, Vogelstein B, Zhang L (2003). PUMA mediates the apoptotic response to p53 in colorectal cancer cells. Proc Natl Acad Sci U S A.

[165] Zaccara S, Tebaldi T, Pederiva C, Ciribilli Y, Bisio A, Inga A (2014). p53-directed translational control can shape and expand the universe of p53 target genes. Cell Death Differ.

[166] Cuadrado A, Lafarga V, Cheung PC, Dolado I, Llanos S, Cohen P (2007). A new p38 MAP kinase-regulated transcriptional coactivator that stimulates p53-dependent apoptosis. EMBO J.

[167] Wu WS, Heinrichs S, Xu D, Garrison SP, Zambetti GP, Adams JM (2005). Slug antagonizes p53-mediated apoptosis of hematopoietic progenitors by repressing puma. Cell.

[168] Schlegel CR, Fonseca AV, Stocker S, Georgiou ML, Misterek MB, Munro CE (2014). DAPK2 is a novel modulator of TRAIL-induced apoptosis. Cell Death Differ.

[169] Flores ER, Tsai KY, Crowley D, Sengupta S, Yang A, McKeon F (2002). p63 and p73 are required for p53-dependent apoptosis in response to DNA damage. Nature.

[170] Petre-Lazar B, Livera G, Moreno SG, Trautmann E, Duquenne C, Hanoux V (2007). The role of p63 in germ cell apoptosis in the developing testis. J Cell Physiol.

[171] Bunjobpol W, Dulloo I, Igarashi K, Concin N, Matsuo K, Sabapathy K (2014). Suppression of acetylpolyamine oxidase by selected AP-1 members regulates DNp73 abundance: mechanistic insights for overcoming DNp73-mediated resistance to chemotherapeutic drugs. Cell Death Differ.

[172] Busuttil V, Droin N, McCormick L, Bernassola F, Candi E, Melino G (2010). NF-kappaB inhibits T-cell activation-induced, p73-dependent cell death by induction of MDM2. Proc Natl Acad Sci U S A.

[173] Montero J, Dutta C, van BD, Weinstock D, Letai A (2013). p53 regulates a non-apoptotic death induced by ROS. Cell Death Differ.

[174] Lake RJ, Basheer A, Fan HY (2011). Reciprocally regulated chromatin association of Cockayne syndrome protein B and p53 protein. J Biol Chem.

[175] Erol A (2011). Genotoxic stress-mediated cell cycle activities for the decision of cellular fate. Cell Cycle.

[176] Macia A, Vaquero M, Gou-Fabregas M, Castelblanco E, Valdivielso JM, Anerillas C (2014). Sprouty1 induces a senescence-associated secretory phenotype by regulating NFkappaB activity: implications for tumorigenesis. Cell Death Differ.

[177] Dong CM, Wang XL, Wang GM, Zhang WJ, Zhu L, Gao S (2014). A stress-induced cellular aging model with postnatal neural stem cells. Cell Death Dis.

[178] Wilhelm MT, Rufini A, Wetzel MK, Tsuchihara K, Inoue S, Tomasini R (2010). Isoform-specific p73 knockout mice reveal a novel role for delta Np73 in the DNA damage response pathway. Genes Dev.

[179] Petrenko O, Zaika A, Moll UM (2003). deltaNp73 facilitates cell immortalization and cooperates with oncogenic Ras in cellular transformation in vivo. Mol Cell Biol.

[180] He Z, Liu H, Agostini M, Yousefi S, Perren A, Tschan MP (2013). p73 regulates autophagy and hepatocellular lipid metabolism through a transcriptional activation of the ATG5 gene. Cell Death Differ.

[181] Su X, Flores ER (2009). TAp63: The fountain of youth. Aging (Albany NY).

[182] Su X, Paris M, Gi YJ, Tsai KY, Cho MS, Lin YL (2009). TAp63 prevents premature aging by promoting adult stem cell maintenance. Cell Stem Cell.

[183] Guo X, Keyes WM, Papazoglu C, Zuber J, Li W, Lowe SW (2009). TAp63 induces senescence and suppresses tumorigenesis in vivo. Nat Cell Biol.

[184] Candi E, Dinsdale D, Rufini A, Salomoni P, Knight RA, Mueller M (2007). TAp63 and DeltaNp63 in cancer and epidermal development. Cell Cycle.

